# Prescription opioid misuse in people with chronic noncancer pain: A multi-variable analysis of sociodemographic, clinical, and psychological factors

**DOI:** 10.1371/journal.pone.0334918

**Published:** 2025-10-22

**Authors:** Carmen Ramírez-Maestre, Rosa Esteve, Elena R. Serrano-Ibáñez, Alicia E. López-Martínez

**Affiliations:** 1 Universidad de Málaga, Facultad de Psicología y Logopedia, Andalucía Tech, Málaga, Spain; 2 Instituto de Investigación Biomédica de Málaga y Plataforma en Nanomedicina (IBIMA Plataforma BIONAND), Málaga, Spain; Jawaharlal Institute of Postgraduate Medical Education and Research, INDIA

## Abstract

Previous research has identified associations between sociodemographic, clinical, and psychological factors and prescription opioid misuse in individuals with chronic noncancer pain (CNCP). A two-study design was used to identify the factors with the most robust association with prescription opioid misuse (Study 1) and to cross-validate these associations in a second sample of people with CNCP to select a reduced number of variables (Study 2). Study 1 included 187 people with CNCP. Point biserial and bivariate correlations, and chi-square analysis showed that the variables significantly associated with opioid misuse were impulsiveness, anxiety sensitivity (AS), pain acceptance, pain catastrophizing, anxiety, depression, posttraumatic stress symptoms (PTSD), and social desirability (medium effect sizes). A family history of alcohol/drug abuse and being between 16 and 45 years of age also reached statistical significance. Study 2 included 179 people with CNCP. The results corroborated the associations found between opioid misuse and impulsiveness, AS, pain acceptance, pain catastrophizing, anxiety, depression, and PTSD. Logistic regression showed that AS, PTSD, and pain acceptance contributed significantly to the unique variance in prescription opioid misuse. Therefore, when prescribing opioids, clinicians should increase the supervision of those people with high AS, PTSD, and low pain acceptance. Evaluating these three variables in people with CNCP who are eligible for opioid therapy could aid in their therapeutic management and help prevent possible iatrogenic effects of opioids. Likewise, this could significantly enhance individual well-being and help mitigate the social problem associated with the misuse of prescription opioids.

## Introduction

Chronic non-cancer pain (CNCP) is defined as pain that persists or recurs for more than 3 months and is not caused by cancer. The most common types of CNCP include musculoskeletal pain (e.g., low back pain, fibromyalgia), neuropathic pain (e.g., trigeminal neuralgia), functional pain syndromes (e.g., irritable bowel syndrome), chronic postsurgical pain (e.g., chronic pain after spinal surgery), and complex regional pain syndrome (e.g., Reflex Sympathetic Dystrophy) [1]. It is a global health problem and a leading cause of medical consultations, disability, and healthcare costs [1]. Among the main therapeutic approaches to its management is the prescription of opioids [2]. While meta-analyses demonstrate the efficacy of this approach (e.g., [3]), other studies also highlight the risks associated with this treatment [4]. Following the publication of clinical guidelines for prescribing opioids [5], a decrease in prescriptions for these drugs has been observed in the United States; however, there has been a notable increase in in opioid prescriptions in Europe in recent decades [6]. This has been particularly evident in Spain, where the defined daily dose of opioids per thousand inhabitants (DDD) has almost doubled from 2015 to the present day [7]. One of the most common adverse effects of long-term opioid treatment for chronic pain is opioid misuse [4]. This is defined as using opioids other than those prescribed, including overuse [8]. A recent meta-analysis showed that the misuse of prescription opioids is relatively frequent among patients with chronic pain undergoing opioid therapy. Estimates indicate that approximately 10% meet criteria for opioid dependence or use disorder, around 33% present with clinical signs or symptoms suggestive of these conditions, and nearly 20% demonstrate aberrant drug-related behaviors [9]. Nearly half of patients receiving treatment for opioid use disorder began by misusing a prescription pain management medication [10].

The scientific literature identifies multiple variables associated with opioid misuse in people with CNCP. These variables include sociodemographic and clinical factors such as age, obesity, smoking, benzodiazepine use, a family or personal history of substance abuse, and duration of opioid treatment [11–15]. Psychological variables have also been shown to contribute to opioid misuse [8]. A positive association has been found between diagnoses of anxiety, depression, and other serious mental illnesses, and opioid misuse [16]. Similarly, experiencing sexual abuse in childhood or suffering from posttraumatic stress disorder (PTSD) appears to increase the risk of developing an opioid use disorder after exposure to opioid analgesics [15,17–19]. Regarding PTSD, a review indicated that 18% of people who use opioids also experience this disorder [20]. Furthermore, prescription opioid misuse is frequently reported among people with coexisting chronic pain and PTSD [21]. An association has also been found between pain catastrophizing, or the anticipation of uncontrollable negative outcomes, and opioid misuse [8,22,23]. However, other studies (e.g., [24]) have not found this association. Instead, their results suggest that pain acceptance is the variable associated with opioid misuse, with anxious-depressive symptoms mediating this relationship [24]. As a process within the psychological flexibility model [25], acceptance involves dealing with stressful experiences without attempting to control or avoid them, while continuing to engage in meaningful daily activities despite their presence [26]. Furthermore, pain acceptance involves coming into direct contact with unpleasant experiences, such as pain sensations or pain-related thoughts and emotions, without attempting to control or avoid them [27]. Several studies have found significant positive associations between pain non-acceptance and opioid misuse (e.g., [28]).

When analysing which transdiagnostic psychological variables could be involved in opioid misuse, studies have found that anxiety sensitivity (AS; fear of anxiety-related physical sensations) or impulsiveness (poorly thought out, hasty, excessively risk or inappropriate actions that often lead to undesirable consequences) could be contributing factors [28–31]. The fear-avoidance model includes AS as a variable present in individuals prior to pain onset [32]. A recent investigation [28] found that impulsivity and AS are antecedent factors that may predispose individuals with CNCP to poor adaptation to chronic pain and prescription opioid misuse. On the other hand, Zvolensky et al. [14] showed indirect effects of PTSD severity via AS in relation to opioid misuse. Furthermore, associations have also been found between impulsiveness (i.e., poorly thought out, hasty, excessively risky, or inappropriate actions that often lead to undesirable consequences) and prescription opioid misuse in individuals with CNCP [31].

Finally, previous research has also shown that social desirability influences self-reported substance abuse [33]. These authors found an association between higher levels of social desirability and a lower frequency of self-reports of abuse and/or opioid misuse in individuals with CNCP. They also suggested that the social stigma associated with opioid abuse and misuse could explain this result [33].

In summary, numerous studies have examined the relationship between sociodemographic, clinical, and psychological factors and prescription opioid misuse in people with chronic pain [19]. However, most research has focused on the role of a limited number of these variables in isolation. Notably, many of these factors are closely related, making it difficult to determine the nature of their associations with prescription opioid misuse. Therefore, confounding may occur when investigating associations between a single factor and an outcome variable, such as opioid misuse, as it remains unclear whether the association arises from the factor analysed or from a related variable that has not been considered [34].

This study aimed to identify those factors with the most robust association with prescription opioid misuse (Study 1) and cross-validate the associations found in a second sample in order to select a reduced number of variables (Study 2).

## Study 1: Identify the variables with the most robust association with opioid misuse

### Materials and methods (Study 1)

We hypothesized that there would be a significant positive association between prescription opioid misuse and being aged between 16 and 45 years (hypothesis 1, H1), body mass index greater than 30 (H2), a history of smoking and being a current smoker (H3), time in treatment with opioids (H4), benzodiazepine use (H5), [11–14]; depressive symptoms (H6), anxious symptoms (H7), serious mental illness (H8), [16], history of child sexual abuse (H9) [18], severity of PTSD symptoms (H10) [17–20]; personal or family history of substance abuse (H11) [15]; AS (H12), impulsiveness (H13) [28–31], and pain catastrophizing (H14) [22–23]. Additionally, we hypothesized that there would be a significant negative association between pain acceptance (H15) [24–27], social desirability (H16) [33], and opioid misuse.

#### Participants.

The required sample size was estimated using GPower 3.1 software for a linear multiple regression analysis, aiming to detect a medium effect size (Cohen’s f² = 0.15) with a statistical power of 0.80 and an alpha level of 0.05 (two-tailed). Based on a model including 15 predictor variables, the analysis indicated that a minimum of 139 participants would be necessary to detect statistically significant relationships with 95% confidence. This estimation aligns with Green’s [35] recommendation for regression models, which suggests a minimum sample size of n ≥ 50 + 8m* (where m is the number of predictors), yielding n ≥ 170 in this case. Therefore, a target sample size of at least 139–170 subjects was considered appropriate to ensure sufficient statistical power and model

Additionally, according to Freeman [36], the sample size required for logistic regression analysis must be [n = 10 * (k + 1)] (where k = number of variables). This study required at least 160 participants [10 * (15 + 1) = 160].

The Study 1 participants comprised a consecutive sample of 187 individuals with CNCP from Spain (Europe) who attended 3 pain units of general hospitals. Inclusion criteria were as follows: participants had to be experiencing CNCP at the time of the study and had been receiving pharmacological treatment that included opioid analgesics for a minimum of 3 months; additionally, they were required to have sufficient proficiency in the Spanish language to understand the study procedures. Exclusion criteria included current treatment for a malignancy, the presence of a terminal illness, or the diagnosis of a severe psychiatric disorder (e.g., schizophrenia, psychotic disorders) in the acute phase, as determined by the mental health unit, which could interfere with study participation.

#### Procedures (Study 1 and 2).

This research (Study 1 and 2) is part of a research project that was approved by the Research Ethics Committee of the Province of Andalusia (PEIBA; CEIP-281021). Written informed consent was obtained prior to data collection. We confirm that all research was performed in accordance with relevant guidelines/regulations.

At the end of the appointment with their pain physician, each patient who was believed by the pain specialist to meet the eligibility criteria was informed of the study aims and their participation was requested. Three psychologists contacted the participants by telephone, assessed their eligibility based on the inclusion criteria, and scheduled appointments with those who consented to participate in the study. Each participant took part in a semi-structured interview with a psychologist from the research team to obtain sociodemographic variables and medical history data. A battery of questionnaires, which measured the variables that we expected to be related to the misuse of prescription opioids, was also completed by each participant in this assessment session. The interviews took about 90 min on average to complete. Neither the participants nor their referring physicians received any economic compensation for study participation. The patients were always assessed in their usual health centre (pain unit).

#### Measures.

*Opioid Risk Tool:* The Opioid Risk Tool (ORT-10; [37,38]) was used to evaluate family and personal history of substance abuse, age, a history of preadolescent sexual abuse, and specific psychological disorders (i.e., having a diagnosis of attention deficit disorder, obsessive-compulsive disorder, bipolar disorder, schizophrenia, or depression). The ORT-10 is a 10-item scale that assesses these five domains or factors associated with prescription opioid misuse.

*Barratt Impulsiveness Scale:* The 30-item Barratt Impulsiveness Scale for adults (BIS-11; [39–41]) was used to measure impulsiveness. Items describing different forms of impulsiveness are rated on a 4-point scale ranging from 1 (“Rarely or never”) to 4 (“Always”). Higher total scores indicate higher levels of impulsiveness. Internal consistency in our sample was adequate (Study 1: α = .79; Study 2: α = .70).

*Anxiety Sensitivity Index:* The 18-item Anxiety Sensitivity Index (ASI-3; [42–44]) was used to measure the severity of AS symptoms. Items are rated on a 5-point scale ranging from 0 (“Very little”) to 4 (“Very much”). If the total score is equal to or greater than 23, the respondents are classified as having a high level of AS [40]. Internal consistency in the present study was excellent (Study 1: α = .92; Study 2: α = .93).

*Posttraumatic Stress Disorder Checklist for DSM-5:* The Posttraumatic Stress Disorder Checklist for DSM-5 (PCL-5; [44]) was used to assess PTSD as described in DSM-5. This instrument comprises a 20-item self-report checklist which is rated on a 5-point Likert-scale (ranging from 1 = never to 5 = very often) to indicate the degree to which each symptom had been experienced by the participant over the past month. Internal consistency in the present study was excellent (Study 1: α = .93; Study 2: α = .90).

*Pain Catastrophizing Scale:* The 13-item Pain Catastrophizing Scale (PCS; [46,47]) assesses the degree to which respondents experience various catastrophizing-related thoughts and feelings when in pain. Respondents indicate how often they have such thoughts on a 5-point scale ranging from 1 (“Never”) to 4 (“Always”). Although the items can be scored to measure three components of catastrophizing (i.e., rumination, magnification, and helplessness), we used the total score, which is the one most often used in research. Internal consistency in our sample was excellent (Study 1: α = .91; Study 2: α = .92).

*Chronic Pain Acceptance Questionnaire:* We administered the 20-item of the Chronic Pain Acceptance Questionnaire (CPAQ-SV; [48,49]). The items on the CPAQ can be scored on either of two subscales (Pain Willingness and Activity Engagement) or on both. The former subscale assesses a general willingness to experience pain, and the latter subscale measures engagement in activities despite pain (i.e., the opposite of pain interference). Respondents rate how often they experience pain willingness and activity engagement on a 7-point scale ranging from 0 (“Never”) to 6 (“Always”). The CPAQ- SV is similar to the original scale in that it yields a total score and two subscale scores for Pain Willingness and Activity Engagement. This study only used the total score. Internal consistency in our sample was good (Study 1: α = .89; Study 2: α = .84).

*Hospital Anxiety and Depression Scale:* The Hospital Anxiety and Depression Scale (HADS; [50,51]) is a self-reporting scale that contains two 7-item subscales: one measures anxiety and the other measures depression. These subscales are scored on a 4-point scale ranging from 1 (“Rarely”) to 4 (“Often”). This study used both subscales. Internal consistency of both scales in our sample was high (Study 1: α = .85 for anxiety; α = .86 for depression; Study 2: α = .83 for anxiety; α = .85 for depression).

*Numerical rating scale:* Participants were asked to rate their current pain intensity, as well as their least, average, and worst pain intensity during the past 2 weeks, on a numerical rating scale ranging from 0 (“No pain”) to 10 (“Pain as intense as you could imagine”). A composite measure of characteristic pain intensity was computed for each participant by calculating the average of the least, average, worst, and current pain ratings. Such composite scores have been shown to be very reliable in individuals with chronic pain [52]. The internal consistency of the composite score in our sample indicated good reliability (Study 1: α = .83; Study 2: α = .80).

*Opioid intake doses:* To assess prescribed opioid intake, we asked participants about their current pain medications, dosages, and frequency of intake. We then computed morphine milligram equivalents (MME) using the methods recommended by Dowell and colleagues [53]: MME/day.

*Current Opioid Misuse Measure:* The current misuse of prescribed opioids was assessed by means of the Current Opioid Misuse Measure (COMM; [54,55], which is a brief self-assessment measure developed to monitor chronic pain patients on opioid therapy. The COMM comprises 17 items asking participants to rate how often they engaged in opioid misuse behaviour in the past 30 days on a scale ranging from 0 (“Never”) to 4 (“Very often”). The items are then summed to create a total score. A total score of 9 or more is considered to be an indication of high risk for opioid misuse. Using this cut-off point, the variable is analysed as dichotomous (misuse/no misuse). However, following the findings of Rogers et al. [56], the COMM total score is also used. Therefore, both the COMM total score and the cut-off point were used in the current study. In our samples, the internal consistency of the measure was good (Study 1: α = .84; Study 2: α = .75).

*Marlowe-Crowne Social Desirability Scale:* To evaluate the potential impact of social desirability bias on response, the short version of the Marlowe-Crowne Social Desirability Scale (MCSDS; [57]) was used*.* This instrument measures the tendency of participants to give answers according to social norms. The scale consists of 18 dichotomous items (yes/no) and showed high internal consistency (α between 0.72 and 0.80). In sample 1, Cronbach’s alpha value was low but similar to those obtained in previous studies [57]: α = .70. However, in sample 2 Cronbach’s alpha value did not reach an acceptable level (α = .65).

#### Statistical Analysis.

In both studies, descriptive statistics were generated for the demographic, clinical, and other variables to describe the samples and studies variables.

The potential factors that have been identified in the literature were evaluated for potential univariate associations with opioid misuse. We treated opioid misuse as both a continuous and a dichotomous variable and computed bivariate correlation coefficients (to analyse the strength and direction of the relationship between two continuous variables) and the point biserial correlation coefficients (to analyse the strength and direction of the relationship between a continuous variable and a dichotomous variable) to test its association with the continuous hypothesised variables. Correlations can be interpreted following the guidelines proposed by Cohen [58]; however, these are only guidelines and should be adapted according to the hypothesis and goals of the study [59]. Furthermore, it must be taken into account that a high point-biserial correlation is smaller than what might be considered to be a high Pearson correlation between two quantitative variables [60]. Chi square tests and contingency analysis were used to test the association between opioid misuse and categorical variables. In order to know how strongly each categorical variable was associated with opioid misuse, Cramer’s *V* was calculated as an effect size measurement for the chi-square test of independence. As Cohen [58] suggested, for chi-square tests with degrees of freedom equal to 2, a value of Cramer’s *V* within the range of.07–.21 indicates a small effect, a value ranging from.21 to.35 indicates a medium effect, and a value equal to or more than.35 indicates a large effect [58,61]. Missing values were replaced with the mean of the available data.

In both studies, all data were analysed using the SPSS 22.0 software package for Windows (SPSS Inc., Chicago, IL).

### Results (Study 1)

#### Participants.

**Fig 1:**
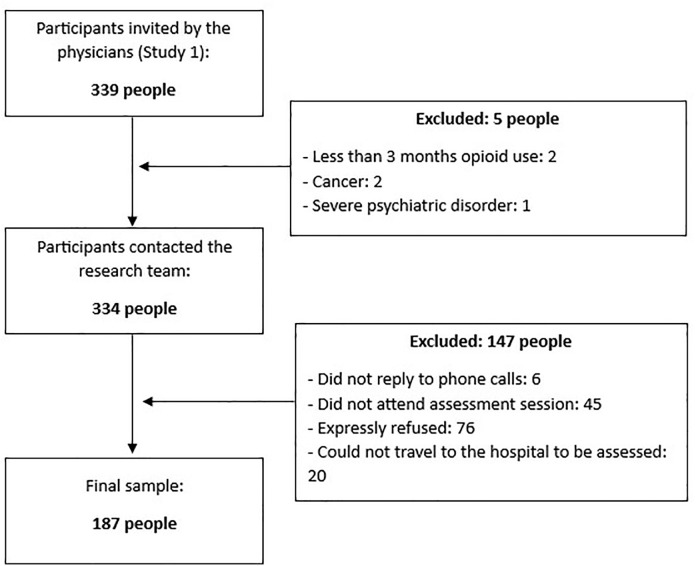
Recruitment of Study 1 participants.

The Study 1 participants comprised a consecutive sample of 187 individuals with CNCP from Spain (Europe; same race and ethnicity) who were recruited while attending clinical consultations in 3 outpatient pain units of general hospitals (see Fig 1). The recruitment process lasted from May 2021 to July 2022.

Table 1 shows the participants’ demographic and clinical characteristics.

**Table 1:** Demographic and Clinical characteristics of the study sample (N = 187).

*Variables*	Mean	SD	Min/max
Age (years) Time in pain (months) Pain Intensity Daily MME/day Time in treatment with opioids (months)	56.1 177.4 6.6 58.1 46.8	11.6 146.1 1.6 78.1 50.9	24/85 10/636 1/10 1.6/530 3/300
	N	%	
**Sex** Men Women	64 123	34 66	
**Marital status** Single Married/Unmarried couple Divorced/Separated Widowed **Education** Reading and writing Primary school High school University education	20 124 31 12 13 48 92 34	11 66 17 6 7 26 49 18	
**Work Status** Housekeeping Working Studying Unemployed Retired	6 48 2 49 82	3 26 1 26 44	
**Site of** **Pain** Head Neck Chest Shoulders Back (Lumbar) Arms Hands Hips Thoracic Legs Feet	78 103 40 110 151 91 94 103 81 139 92	42 55 21 59 81 49 50 55 43 74 49	
**Diagnosis** Functional pain syndromes (Fibromyalgia) Complex regional pain syndrome Primary musculoskeletal pain Post-surgical/ Post-traumatic Neuropathic pain Orofacial pain/Headaches Musculoskeletal pain	55 3 14 23 14 1 77	29 2 8 12 8 1 41	
**Medication use** **Prescribed opioids** Tramadol Tramadol + Dexketoprofen Tramadol + Paracetamol Tapentadol Fentanyl transdermal patch Fentanyl sublingual tablets Oxycodone Oxycodone hydrochloride Buprenorphine transdermal patch Morphine Codeine **Adjuvant medications for treating pain** Benzodiazepines Antidepressants Antiepileptic drugs Hypnotics	47 3 37 63 16 5 4 27 4 11 4 136 123 102 12	25 2 20 34 9 3 2 14 2 6 2 73 66 55 6	

Note. MME: Morphine Milligram Equivalents

MME= 58.1 is considered a moderate dose (Dowell et al., 2016)

In total, 59% of the participants had a score of more than 9 on the COMM, which indicates prescribed opioid misuse, and 73% of the participants had a prescription for benzodiazepines. Mean pain duration was 15 years (mean = 177.4 months; SD = 146.1), and mean time on opioids was 47 months (SD = 50.5).

#### Descriptive and correlation analyses.

Table 2 shows the means, standard deviations, and point biserial correlation coefficients between continuous antecedent variables and the dichotomous outcome variable *misuse/non-misuse*. The variable Social Desirability was included in this analysis. To be conservative in this first study, and considering that a high point-biserial correlation is smaller than what might be considered to be a high Pearson correlation between two quantitative variables [60], variables with a significant correlation coefficient equal to or greater than.20 in point biserial correlation and equal to or greater than.30 in Pearson bivariate correlations were included in the Study 2 analysis.

**Table 2:** Means, Standard Deviations, and Correlations between the model variables (Sample 1: N = 187).

	Min/max	M	SD	1	2	3	4	5	6	7	8	9	10	11
1. Impulsiveness	6/84	42.5	16.5	1										
2. Anxiety Sensitivity	0/72	18.7	16.6	.28**	1									
3. PTSD symptoms	0/77	38.8	15.8	.40**	.40**	1								
4. Catastrophizing	13/52	36.3	10.3	.16	41**	.24**	1							
5. Acceptance	4/110	47.1	25.2	−.16*	−.32**	−.20*	−.64**	1						
6. Pain	1/10	6.6	1.6	.07	.23**	.15	.29**	−.24**	1					
7. Anxiety	7/28	18.3	9.1	.44**	.44**	.50**	.51**	−.39**	.33**	1				
8. Depression	7/28	16.2	5.9	.36**	.35**	.33**	.46**	−.63**	.26**	.65**	1			
9. Social Desirability	3/18	11.1	3.3	−.32**	−.25**	−.14	−.24**	.18*	−.04	−.13	−.23**	1		
10. Time in treatment with opioids (months)	3/300	46.7	50.5	−.00	−.02	.07	−.00	−.05	.09	.10	.09	.15*	1	
11. Misuse* (continuous)	0/52	13.2	9.3	.37**	.42**	.39**	.38**	−.38**	.21**	.40**	.46**	−.43**	**.04**	**1**
12. Misuse** (Dichotomous)				.37**	.33**	.22**	.29**	−.31**	**.13**	.31**	.35**	−.32**	**.06**	.70**

* Correlations between opioid misuse (continuous variable) and the other continuous variables

** Biserial correlations between opioid misuse (dichotomous variable) and the continuous variables

Note. Significance level: ** *p* < .001: * *p* < .05

The associations between the variables and opioid misuse were in the directions indicated in the hypotheses (H4, H6-7, H10, and H12-16) and most of the associations reached statistical significance (*p* ≤ .001). Except for time in treatment with opioids, all the variables assumed to be associated with opioid misuse showed significant associations. In the Pearson bivariate correlation analysis, pain intensity showed a low effect size but significant associations with opioid misuse (r < .30). Impulsiveness, AS, PTSD, pain catastrophizing, pain acceptance, anxiety and depression symptoms, and social desirability showed medium effect sizes and significant associations with opioid misuse (*r* range from.37 to.46). In point biserial correlation, the association between pain intensity and opioid misuse was not significant. Impulsiveness, AS, pain acceptance, anxiety and depression symptoms, and social desirability showed medium effect sizes and significant associations with opioid misuse (*r*_*pb*_ range from.31 to.36), while PTSD symptoms and pain catastrophizing showed lower but significant associations (*r*_*pb*_ = .22 and.29).

#### Chi-square analysis.

The association between categorical antecedent variables and opioid misuse were tested with a Chi square tests and contingency analysis. Cramer’s *V* was calculated to determine how strongly each categorical variable was associated with opioid misuse. Again, to be conservative in this first study, those variables whose association with opioid misuse obtained a value of Cramer’s *V*’s equal to or greater than.20 were included in the analysis of Study 2. Furthermore, to avoid Type I errors, the significance level was adjusted to.006 or below. According to the hypothesis (H1, H3, H5, and H11), having a family history of alcohol abuse, a family history of drug abuse, a personal history of medication abuse, being between 16 and 45 years of age, a history of smoking and being a current smoker, and taking benzodiazepines or antidepressants were significantly associated with opioid misuse. However, only a family history of alcohol abuse, a family history of drug abuse, and being between 16 and 45 years of age reached satisfactory level of significance and Cramer´s *V* size (≥.20). The results did not confirm hypotheses H2, H5, and H8. Table 3 shows the results of Chi-square analysis.

**Table 3:** Frequencies and Chi-quare results in relation to categorical antecedent factors (*N* = 187).

Antecedent Variables	Sample 1		*χ* ^*2*^	*p*	Cramer’s *V*
	*n*	*%*			
Family history of alcohol abuse	69	37	7.78	.006	.20
Family history of medication abuse	15	8	0.01	.999	.04
Family history of drug abuse	36	19	8.30	.004	.21
Personal history of alcohol abuse	11	6	0.86	.529	.07
Personal history of medication abuse	13	7	6.28	.016	.18
Personal history of drug abuse	16	9	3.47	.068	.13
Being between 16 and 45 years of age	30	16	13.91	.000	.23
History of preadolescent sexual abuse	34	18	2.17	.177	.11
Psychological disorder (ADD, OCD, bipolar, schizophrenia)	3	2	2.08	.272	.11
History of smoking and being a current smoker	61	33	4.65	.039	.16
Body mass index more than 30	59	32	0.54	.515	.06
Sex (women) Benzodiazepine intake Antidepressant intake	123 136 123	66 73 66	2.45 4.83 3.46	.158 .040 .081	.11 .16 .14

*Note.* Frequencies and percentages indicate the presence of the characteristic (marked as a “yes” answer).

Therefore, in line with these results, the continuous variables that had to be included in Study 2 were as follows: Impulsiveness, AS, PTSD, anxiety and depression symptoms, pain catastrophizing, pain acceptance, social desirability and, regarding the categorical variables, having a family history of alcohol abuse, a family history of drug abuse, and being between 16 and 45 years of age.

## Study 2. cross validation

### Materials and methods (Study 2)

#### Participants.

**Fig 2:**
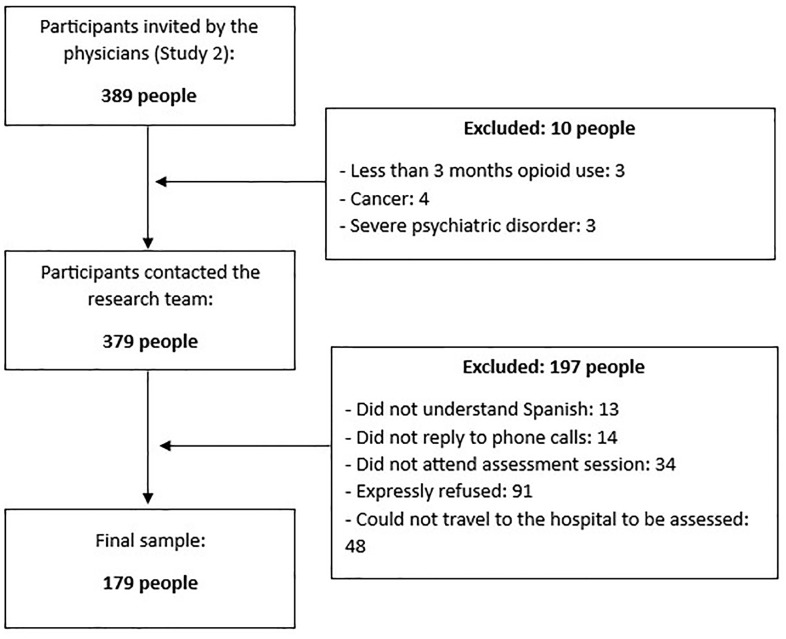
Recruitment of Study 2 participants.

The participants of Study 2 consisted of 179 individuals who shared the same characteristics as those in Study 1 (individuals with CNCP from Spain who were recruited while attending clinical consultations in 3 pain units of general hospitals). Likewise, the inclusion and exclusion criteria were identical to those previously described (see Fig 2).

#### Procedures.

The procedures used were the same as those carried out in Study 1.

#### Measures.

The same instruments previously described were used to evaluate the variables of Study 2. The ORT-10 [37,38] was used to assess a family and personal history of substance abuse, and age. The BIS-11 [39–41] was used to measure impulsiveness. The ASI-3 [42,43] was used to measure the severity of AS symptoms. The PCL-5 [45] was used to assess PTSD. The PCS [46,47] was used to assess the degree to which respondents experience various catastrophizing-related thoughts and feelings when in pain. We administered the CPAQ-SV [48,49] to measure pain acceptance. Anxiety and depressions symptoms were measured with the HADS [49,50]. Again, a composite measure of characteristic pain intensity was computed for each participant by calculating the average of the least, average, worst, and current pain ratings. To assess prescribed opioid intake, we computed morphine milligram equivalents (MME) using the methods recommended by Dowell et al. [53]: MME/day; the current misuse of prescribed opioids was assessed by means of the COMM [54,55]. Finally, the MCSDS [57] was used to evaluate the potential impact of social desirability bias on responses.

#### Statistical analysis.

Descriptive statistics were generated for the demographic, clinical, and other variables to describe the samples and study variables. According to the results obtained in Study 1, we modified the initial model of relationships, excluding all those variables that were not significantly associated with opioid misuse.

Firstly, we computed the Pearson correlation coefficients (COMM total score as a continuous variable) and the point biserial correlation coefficients (COMM as a dichotomous variable) between the variable opioid misuse and the psychological variables hypothesised as associated with prescription opioid misuse, according to the results of Study 1. In Study 2, to account for multiple comparisons, we applied a Bonferroni correction to the correlation analyses. Given that nine variables were tested, the significance threshold was adjusted to p < .0056 (.05/9). This conservative approach was used to reduce the likelihood of Type I errors and ensure the robustness of the observed associations. Again, Chi square tests and contingency analysis were used to test the association between opioid misuse and categorical variables, and Cramer’s *V* was calculated as an effect size measurement for the chi-square test of independence.

Next, we performed a linear regression to analyse potential multicollinearity between the variables related to opioid misuse. The Variance Inflation Factor (VIF) was used as a measure of potential multicollinearity between the variables. There is no formal cutoff value for the VIF to determine the presence of multicollinearity [62]. In logistic regression, values of VIF above 2.5 may be of concern [63] and it is advisable to remove some of the variables causing multicollinearity from the model [62]. Consequently, we conducted a binomial logistic regression with opioid misuse as the response variable, including only the antecedent variables with a VIF value less than 2.5 in the regression model. Therefore, linear and binomial logistic regression analyses were used to identify significant associations between the set of variables included in the hypothesis and opioid misuse. The following step was taken to fit a parsimonious model with only strong predictive covariates. Binomial logistic regression was used to identify the combined effect of several factors. Using a forward stepwise procedure, the variables that achieved a *p* value .05 remained in the final model. The results are expressed as odds ratios (ORs) with 95% confidence intervals (CIs) and p values. A two-tailed P value of <.05 was used as a cutoff for statistical significance.

### Results (Study 2)

#### Participants.

The recruitment process lasted from October 2022 to June 2023.

Table 4 shows the participants’ demographic and clinical characteristics.

**Table 4:** Demographic and clinical characteristics of the study sample (sample 2: N = 179).

*Variables*	Mean	SD	Min/max
Age (years) Time in pain (months) Pain Intensity Daily MME/day Time in treatment with opioids (months)	60.3 229.9 6.6 53.4 60.7	12.2 190.7 1.4 76.2 59.5	26/84 9/887 2.5/10 0.4/600 6/360
	N	%	
**Sex** Men Women	64 115	36 64	
**Marital status** Single Married/Unmarried couple Divorced/Separated Widowed **Education** Reading and writing Primary school High school University education	23 122 21 13 29 60 68 22	13 68 12 7 16 33 39 12	
**Work Status** Housekeeping Working Studying Unemployed Retired	12 42 1 21 103	7 23 1 12 57	
**Site of** **Pain** Head Neck Chest Shoulders Back (Lumbar) Arms Hands Hips Thoracic Legs Feet **Diagnosis** Functional pain syndromes (Fibromyalgia) Complex regional pain syndrome Primary musculoskeletal pain Post-surgical/ Post-traumatic Neuropathic pain Orofacial pain/Headaches Musculoskeletal pain **Medication use** **Prescribed opioids** Tramadol Tramadol + Dexketoprofen Tramadol + Paracetamol Tapentadol Fentanyl transdermal patch Fentanyl sublingual tablets Oxycodone Oxycodone hydrochloride Buprenorphine transdermal patch Morphine Codeine	102 134 54 128 160 121 124 133 95 154 130 46 2 3 27 12 1 88 40 4 55 33 22 4 3 28 2 7 4	57 75 30 72 89 68 69 74 53 86 73 26 1 2 15 7 1 49 22 2 31 18 12 2 2 16 1 4 2	
**Adjuvant medications for treating pain** Benzodiazepines Antidepressants Antiepileptic drugs Hypnotics	92 113 99 10	51 63 55 6	

Note. MME: Morphine Milligram Equivalents.

MME = 53.4 is considered a moderate dose

In total, 25% of the participants had a score of more than 9 on the COMM, which indicates prescribed opioid misuse. Also, 51% of the participants had a prescription for benzodiazepines. Mean pain duration was 19 years (Mean = 229.9 months; SD = 190.7), and mean time on opioids was 61 months (SD = 59.5)

#### Descriptive and correlation analyses.

Table 5 shows the means and standard deviations of the continuous variables and the point biserial (COMM as a dichotomous variable) and Pearson bivariate (COMM as a continuous variable) correlation coefficients between continuous antecedent variables and the outcome variable *opioid misuse*.

**Table 5:** Means, standard deviations, and correlations between the variables (Sample 2: N = 179).

	Min/max	M	SD	1	2	3	4	5	6	7	8	9
1. Impulsiveness	13/82	43.4	13.3	1								
2. Anxiety Sensitivity	0/69	22.5	15.8	.35**	1							
3. PTSD symptoms	0/77	37.9	17.8	.40**	.58**	1						
4. Catastrophizing	13/52	36.3	10.5	.32**	54**	.47**	1					
5. Acceptance	9/108	52.4	21.1	−.19*	−.39**	−.44*	−.63**	1				
6.Anxiety	7/28	18.8	5.6	.53**	.57**	.72**	.56**	−.49**	1			
7.Depression	7/28	15.7	5.6	.26**	.43**	.64**	.50**	−.64**	.63**	1		
8. Misuse* (continuous)	0/43	13.2	8.4	.33**	.43**	.49**	.45**	−.47**	.60**	.51**	1	
9. Misuse** (Dichotomous)				.23**	.35**	.51**	.36**	−.35**	.49**	.43**	.77**	**1**

* Correlations between opioid misuse (continuous variable) and the other continuous variables

** Biserial correlations between opioid misuse (dichotomous variable) and the continuous variables

Note. Significance level: ** *p* < .001: * *p* < .05

The associations between the continuous variables and opioid misuse were in the expected directions. All the associations reached statistical significance (*p* ≤ .001). Impulsiveness, AS, pain acceptance, pain catastrophizing, PTSD, anxiety and depression symptoms showed medium-high effect sizes and a significant association with opioid misuse (*r* range from.33 to.60; *r*_*pb*_ range from.23 to.51). On the other hand, PTSD, anxiety, and depression symptoms, as well as pain catastrophizing showed high correlations with each other (coefficients of correlation range from.53 to.72). Although the association between social desirability and opioid misuse was significant in Study 1, the reliability of social desirability in Study 2 was not acceptable (α = .65), so this variable was not tested on this occasion.

#### Chi-square analysis.

Table 6 shows the results of Chi-square analysis.

**Table 6:** Frequencies and Chi-quare results in relation to categorical antecedent factors (*sample 2: N = 179*).

Antecedent Variables	Sample 2		*χ* ^*2*^	*p*	Cramer’s *V*
	n	%			
Family history of alcohol/drug abuse	69	39	0.43	.528	.05
Being between 16 and 45 years of age	20	11	6.74	.012	.19

*Note.* Frequencies and percentages indicate the presence of the characteristic (marked as a “yes” answer).

The association between a family history of alcohol/drug abuse and opioid misuse was not significant (*p* = .528). Only the association of age (being between 16 and 45 years of age), and opioid misuse was significant (*p* = .012), although the magnitude of Cramer´s *V* was low (*V* = .19).

#### Regression analysis.

Opioid misuse was included as the outcome variable in the binomial logistic regression. The first step was to fit a parsimonious model with only strong covariates. For this purpose, a linear regression analysis was performed in two steps, introducing the total COMM score as the outcome variable and anxiety sensitivity, impulsivity, catastrophizing, pain acceptance and anxiety, depression, and PTSD symptoms as antecedent variables. The VIF was used as a measure of potential multicollinearity between the variables. Variables with VIF values greater than or equal to 2.5 were removed from the model. Therefore, Anxiety (VIF = 2.6) and depression (VIF = 2.5) symptoms were eliminated from the regression model. The regression analysis was performed in two steps. In step 1, the measures of AS and impulsiveness were entered. In step 2, pain acceptance, pain catastrophizing, and PTSD symptoms were entered. Variables with **p* *≥ .1 were excluded from the equation. Thus, a value of 0.1 was set as the threshold for the removal of variables to reduce the risk of overlooking potential predictors. As shown in Table 7, AS (*β *= .14, **p* *= .05), pain acceptance (*β *= −.31, **p* *< .001), and PTSD (*β *= .29, **p* *< .001) accounted for 36% of the opioid misuse. However, the contributions of impulsiveness (**p* *= .09) and pain catastrophizing (**p* *= .19) were not significant.

**Table 7:** Results of linear regression analysis of variables associated with the misuse of opioid pain medication prescription (COMM total score).

Step and Variables	*Beta (standardized)*	*R* ^*2*^	*F*
Step 1 Anxiety sensitivity Impulsiveness	.35** .21**	.31	27.35**
*Step 2*		.36	26.42**
Anxiety sensitivity	.14*		
Impulsiveness Pain acceptance Pain catastrophizing	.11 *ns* −.31** −.11 *ns*		
PTSD	.29**		

*p <.05; **p <.001; *ns*= non-significant.

Finally, binomial logistic regression was used to identify the combined effect of impulsiveness, AS, pain catastrophizing, pain acceptance, and PTSD. The logistic regression model was statistically significant (χ^2^ = 15.766, *p* = .04) and explained 30% (Nagelkerke *R*^*2*^) of the variance in the criterion variable. The model correctly classified 74% of the cases. AS, pain acceptance, and the presence of symptoms of PTSD contributed significant unique variance to the prediction of the criterion variable; however, impulsiveness (**p* *= .11) and pain catastrophizing (**p* *= .21) were excluded. Table 8 shows the results of the logistic regression analysis.

**Table 8:** Results of logistic regression analysis of variables associated with the prescription opioid misuse.

	*Beta*	*Wald*	*ORs*	*p*	95% C.I.-EXP(B)	
					*low*	*up*
Anxiety Sensitivity Pain Acceptance PTSD	.028 −.028 .035	4.11 8.21 9.28	1.029 .972 1.035	.04 .004 002	1.001 .954 1.012	1.057 .991 1.058

The use of opioid pain medication was coded as 0 = no use and 1 = use.

## Discussion

Delimiting the variables with the most robust association with prescription opioid misuse could help in clinical practice, where time and resources are limited and it is not possible to assess all the potential factors to detect vulnerability to opioid misuse. As hypothesized, anxious symptoms (H6), depressive symptoms (H7), severity of PTSD (H10), AS (H12), impulsiveness (H13), pain catastrophizing (H14), and pain acceptance (H16) were considerably and consistently related to prescription opioid misuse in both samples. However, being aged between 16 and 45 years (H1), body mass index greater than 30 (H2), a history of smoking and being a current smoker (H3), time in treatment with opioids (H4), benzodiazepine use (H5), serious mental illness (H8), history of child sexual abuse (H9), and personal or family history of substance abuse (H11) were not consistently associated with opioid misuse in both studies.

The results of this study showed that AS, pain acceptance, and PTSD symptoms were the variables with the most robust association with prescription opioid misuse. AS is consistently associated with the consumption of drugs that diminish arousal, such as opioids [30,56], since individuals high in AS fear anxiety-related sensations, more precisely bodily sensations, due to beliefs that these sensations will have catastrophic somatic, cognitive, or social consequences [64]. Additionally, there is a known association between AS and anxiety and depression disorders, which are highly prevalent in individuals with chronic pain [65], and between AS and PTSD [66]. At the same time, both the presence of anxiety and depression and PTSD are related to prescription opioid misuse [17,20]. A recent model proposed AS as a transdiagnostic factor accounting for the high comorbidity between anxiety, depression, and opioid misuse and its mutual maintenance [56]. These authors suggested that anxiety, depression, and opioid misuse could share common underlying vulnerabilities and that several factors may modulate these relationships, such as transdiagnostic factors (i.e., AS), trauma exposure, individual differences (i.e., age), and pain experience [56].

Anxiety sensitivity also plays a central role in the adaptation to chronic pain; specifically, in the Fear-Avoidance model of chronic pain [67,68], AS is the predisposition variable antecedent of the processes that lead to disability and depression (pain catastrophizing, fear of pain, hypervigilance, activity avoidance) [68]. Ditre et al. [29] proposed a model where AS played a central role in the mutual maintenance of chronic pain and opioid misuse. Thus, according to this model, AS would contribute to pain chronification through fear of pain and activity avoidance; when opioids are prescribed, they would also contribute to avoiding pain-related internal adverse events as well as opioid-related internal negative events (i.e., withdrawal symptoms).

Pain acceptance is also strongly associated with adaptation to chronic pain [69]. It involves responding to pain-related experiences without attempting to control or avoid them and engaging in valued activities regardless of these experiences [26]. In contrast to avoidance, pain acceptance may represent an adaptive form of “confrontation” [70]. As explained in the introduction section, acceptance is one of the elements of the transdiagnostic model of Psychological Flexibility [25], which considers that medication misuse functions as a form of experiential avoidance guided by the preeminence of the life value of eliminating pain at all costs [27]. There is preliminary evidence that pain acceptance is a protective factor concerning opioid misuse in people with chronic pain [71]. Specifically, Esteve et al. [24] found that pain acceptance was related to opioid misuse through lower anxiety and depression. In this sense, several theoretical models (i.e., [29,72]) have highlighted the role of negative reinforcement as a maintenance factor of addiction problems through a learned association between relief from aversive internal states (negative affect and pain) and opioid intake.

Our results support previous findings regarding PTSD symptoms. As mentioned above, having PTSD increases the risk of developing an opioid use disorder after contact with opioid painkillers [15,17–19]. Several reviews have indicated that 18% of people who use opioids also experience PTSD [20]; additionally, the misuse of prescription opioids is frequently reported among individuals with co-occurring chronic pain and PTSD [21]. Empirical literature has also demonstrated associations between CNCP, opioid misuse, and trauma exposure (e.g., [73]. Individuals who have been exposed to trauma, particularly interpersonal trauma, tend to perceive bodily signals as catastrophic and frightening [74]. Similarly, it has been suggested that individuals attempt to mitigate specific symptoms, including pain, when anxious thoughts are focused on them and they are perceived as uncontrollable [75]. Therefore, the use of opioid medication could represent an attempt to mitigate such symptoms.

Furthermore, anxiety (H6), and depression (H7) symptoms, impulsiveness (H13), and pain catastrophizing (H14) were strongly and consistently associated with prescription opioid misuse in both samples. However, anxiety and depression symptoms were not included in the final regression model to avoid multicollinearity and prevent statistical anomalies [76]. As mentioned above, this is an expected result, since the high co-morbidity among these variables is extensively documented; theoretical models of prescription opioid misuse integrate them through transdiagnostic variables and the mechanisms of adaptation to chronic pain [29].

On the other hand, although the associations of opioid misuse with pain catastrophizing and with impulsiveness were significant in the correlation analyses, they were not significant in the regression models. The empirical literature shows that pain catastrophizing is associated with opioid misuse [8,23]. Additionally, Martel et al. [23] suggested that impulsiveness could mediate the relationship between pain catastrophizing and prescription opioid misuse, while Peck et al. [77] found that impulsiveness underlies both opioid use disorder and PTSD.

In summary, the results of this study do not imply discarding the role of anxiety and depression symptoms, pain catastrophizing, and impulsiveness in prescription opioid misuse, but highlight that when several of the psychological factors associated with opioid misuse are considered together, AS, PTSD, and pain acceptance were the variables with the strongest association.

In contrast to previous research and the study hypotheses (H1-5, H8, H9, H11), neither study found a strong and consistent association between the demographic and clinical variables and prescription opioid misuse. The percentage of benzodiazepine use in the sample was very high. However, although co-occurring benzodiazepine and opioid use has been associated with opioid use disorders [78], no association was found between benzodiazepine and antidepressant use and prescription opioid misuse in our sample. It has to be underlined that, according to previous research, some of these demographic and clinical variables are highly related to anxiety and depression and to the condition of having chronic pain. For example, people with obesity are at higher risk of chronic pain, and are more likely to receive prescription opioids for extended periods [79]. Moreover, people with depressive and anxious disorders will likely use anxiolytics and antidepressants and, interestingly, are more likely to be prescribed opioids and receive higher and stronger doses [80]. Furthermore, opioid and benzodiazepine co-use is more frequent among people with a history of abuse victimization [81].

Regarding the role of pain intensity, when controlling for psychological factors, the relationship between pain intensity and opioid misuse frequently disappears (i.e., [8]).

The association between opioid misuse and several other non-psychological variables, such as a family history of alcohol abuse, a family history of drug abuse, and being between 16 and 45 years of age, reached statistical significance in Study 1. However, in Study 2, these associations did not reach statistical significance, or the strength of the relationships was weak. These factors have been identified as being associated with the increased risk of addictive substance abuse. Some of the most widely studied risk factors include a previous history of addiction (e.g., [18]). Other studies have addressed predictors of abuse in relation to age, with contradictory results [13,38]. All of these variables are included in the Opioid Risk Tool (ORT; [38]), an instrument used to predict the risk of engaging in aberrant drug-related behaviour in patients with chronic pain receiving prescribed opioid therapy. However, the results of Esteve et al. [37] showed that the variables included in the ORT as being associated with opioid misuse were not actually associated with it, with the exception of being between 16 and 45 years of age. Therefore, according to the present and previous results, these variables do not appear to be the most strongly associated with prescription opioid misuse.

In Study 1, as hypothesised, social desirability and self-reported opioid misuse showed a significant negative association (H16). This result is in line with previous research showing that people with a strong desire to project a socially desirable image of themselves are less likely to self-report prescription opioid misuse and abuse and substance misuse and abuse in general [33]. In Study 2, we could not include social desirability in the analyses because of the low reliability of the measure. However, we must always be aware of the potential impact of social desirability on prescription opioid self-reports and take actions to control it.

When interpreting the results of this study, several limitations must be considered. The main limitation of this study is its exclusive reliance on self-report instruments; thus, future research should include more objective measures of those variables. Also, since this is a cross-sectional study, it is not possible to draw causal conclusions regarding the associations between the study variables. Longitudinal methods could be used in future studies. Regarding prescription opioid misuse, when possible, it would be advisable to use blood and urine tests alongside prescription records. Additionally, women were overrepresented; this ratio of women to men was not intentional but is representative of the general population with chronic pain [82]. Furthermore, the sample was relatively homogeneous in terms of sociodemographic characteristics, and all participants were recruited within the Spanish healthcare context. These factors may limit the generalizability of the findings to more diverse populations or to healthcare systems with different cultural, clinical, or regulatory approaches to chronic pain management and opioid prescribing. Future research should aim to replicate these findings in more heterogeneous samples and across different cultural or institutional contexts. Also, missing data were handled by replacing missing values with the mean of the available cases. While this is a simple and commonly used method, it may introduce potential biases, such as underestimating the variance and distorting relationships between variables. Therefore, findings should be interpreted with caution.

Finally, the scientific literature has identified a large number of variables potentially associated with prescription opioid misuse in people with CNCP. Although we have tried to be comprehensive, it is clear that our study cannot cover each and every variable potentially associated with opioid misuse, so we acknowledge that there may be additional variables that have not been analysed. In any case, since each individual interview with the participants lasted between one and a half and two hours, the inclusion of additional variables, and therefore questionnaires, could lead to excessive fatigue among the interviewees. On the other hand, one of the strengths of this study is that we simultaneously followed a comprehensive and parsimonious strategy by first considering numerous well-documented variables associated with prescription opioid misuse in previous research and then reducing them to fewer variables based on their multicollinearity and the strength of their association with opioid misuse.

In summary, this study found that of the many variables associated with prescription opioid misuse in people with chronic pain in previous studies, three stand out: AS, PTSD symptoms, and pain acceptance. These variables are at the core of adaptation to chronic pain and are situated at the poles of avoidance and openness to the negative internal experiences associated with pain. However, the role of impulsivity, pain catastrophizing, and anxiety and depression symptoms in prescription opioid misuse should not be ruled out. In fact, the current results support the development of an instrument that, by evaluating all these variables, could help to identify people vulnerable to opioid misuse. Longitudinal studies will determine whether these variables can effectively predict the risk of opioid misuse in people with CNCP who have started taking opioid medication [83]. As a whole, these results highlight the critical role of psychological variables in opioid misuse. These variables also play a crucial role in the adaptation to chronic pain; thus, when prescribing opioids, clinicians should increase the supervision of those people with high AS, PTSD, and low pain acceptance. At the same time, psychological interventions aimed at promoting adaptation to chronic pain should routinely assess how people with CNCP relate to pain medication and intervene in this respect when necessary.
